# Sleep disturbances among physicians during COVID-19 pandemic

**DOI:** 10.1186/s13104-020-05341-6

**Published:** 2020-10-21

**Authors:** Yasser H. Alnofaiey, Haneen A. Alshehri, Maram M. Alosaimi, Shrooq H. Alswat, Raghad H. Alswat, Rahaf M. Alhulayfi, Meteb A. Alghamdi, Reem M. Alsubaie

**Affiliations:** 1 Department of Internal Medicine, KSA, Taif University, Taif, Saudi Arabia; 2 Medical Student, KSA, Taif University, Taif, Saudi Arabia; 3 Department of Internal Medicine, KAMC, Makkah city, KSA Saudi Arabia

**Keywords:** Sleep, Disturbances, Physicians, COVID-19, Pandemic, Saudi

## Abstract

**Objectives:**

To assess prevalence and related factors of sleep disturbances among Saudi physicians during COVID-19 pandemic. Data were collected through a questionnaire including items about demographic characters, knowledge about covid-19 and items to assess sleep quality that were extracted from Pittsburgh Sleep Quality Index (PSQI) scale.

**Results:**

Prevalence of sleep disorders was 43.9%, doctors in the age group of 31–40 years, associate consultants had a significant higher prevalence of sleep disorders. Medical interns and laboratory/pathology/microbiology doctors had a significant more difficulty in fall asleep during COVID-19, and internists and surgeons had a significant higher percent of those who used sleeping pills. Resident doctors had a significant higher percent of having trouble in staying awake, and residents and consultants had a significant higher percent of those who suffered decreased sleep duration. Sleep quality during COVID-19 was very good, fair good and very bad in 23.4%, 60% and 3.5% of HCW respectively. The study observed a negative impact of COVID-19 pandemic on HCW sleep quality. Hospitals administrations should provide optimal working hours with enough break and employ more doctors during the pandemic. Doctors experiencing sleep problems should have mandatory leaves.

## Introduction

COVID-19 appeared in Wuhan, China, in December 2019 and rapidly spread to become a global pandemic.[1–4]. Clinical manifestations include mainly fever (99%), fatigue (70%), dry cough (60%), myalgia (44%), and dyspnea at the onset of the disease [5, 6]. Treatment of COVID-19 is mostly supportive and no known effective specific treatment exists [7].

All people during COVID-19 pandemic have fears of getting infected or infecting others [8]. Health care workers face other stresses, such as absence of clear strategy to follow and an increased risk of exposure to aerosols [8].

Sleep disorders involve problems either in the quality, timing or amount of sleep [9, 10]. Studies from China identified that healthcare workers were at high risk for poor sleep quality during COVID-19 epidemic [11], and high prevalence of post-traumatic stress syndrome and poor sleep quality [12]. No published studies addressed the problem of sleep quality during COVID-19 epidemic in Saudi Arabia. The current study aimed to assess prevalence and related factors of sleep disturbances among Saudi physicians of the Western province of Kingdom of Saudi Arabia (KSA) during COVID-19 pandemic.

## Main text

### Methods

Study design and setting, and time frame: a cross-sectional survey was carried out in health care settings in the Western province of Saudi Arabia that included the Mecca, Jeddah, and Taif cities. The study was done during the period the period of May 2020 to August 2020.

Study participants: The study was carried out on Saudi doctors who were willing to participate in the survey. Physicians working in other regions of Saudi Arabia and non-Saudi doctors were excluded from our study.

Sample size and sampling method: a minimum sample size of 327 was calculated with 95% confidence level, 5% margin of error and power of study (β-error) at 80% considering the values derived from the pilot study. A mixture of cluster, convenience and snowball sampling was followed to acquire the minimum sample size.

Data collection: a pre-tested and validated questionnaire was used. Social media tools were used to broadcast the relevant link of the questionnaire.

The questionnaire included items to collect demographic data, and knowledge about COVID-19. Knowledge items were about (a) inhalation of droplets from sneezing, coughing, or talking of an infected person (b) contact with something contaminated by an infected person, (c) the incubation period of the virus, (d) contact with an asymptomatic person and possibility of infection, and (e) if there are already targeted drugs that could cure the disease. For all questions responses to items related to knowledge regarding COVID-19 were recorded and each correct response were given a score of ‘1’ and wrong answers were given score ‘0’ (11). Thus, the maximum score one could get is 6. The scores were then categorized based on the percentages into ‘Good’ (80–100%), Fair (60–79%) and Poor (< 59%).

For assessing the sleep quality, five items were extracted from Pittsburgh Sleep Quality Index (PSQI) scale [13], and these include: Subjective sleep quality, difficulty in starting sleep within 30 min, easy waking during sleep and early waking in the morning, use of sleep medication, and the sleep duration, within 1-month lasted. A very good quality sleep is defined when the subject experiences all of the five characteristics mentioned: (a) Sleeping more time while in bed (at least 85% of the total time in bed), (b) Falling asleep in 30 min or less, (c) Sleeping for more 7 h or more, (d) Falling back asleep within 20 min after getting up, and (e) Waking up—for five minutes or longer—no more than once a night. Subjects were asked to rate the quality of their sleep based on the above five points as ‘very good’, ‘fairly good’, ‘fairly bad’ and ‘very bad’. Single-item sleep measures have been used in previous studies [12, 14].

Validation of the Questionnaire: the questionnaire included items that covered knowledge regarding Covid 19 infection and measured the sleep quality. The items related to sleep quality was adopted from Pittsburgh Sleep Quality Index (PSQI) scale by Buysse et al. [13]. Validation of this questionnaire was done using focus group discussion, expert evaluation, pilot study, reliability and validity assessment etc. Three experts in the field of COVID 19 and one biostatistician were involved in the validation of our questionnaire. A pilot study was done on 15 participants and the data obtained was used for reliability and validity analysis. Content validity, face validity, and construct validity of the developed questionnaire were examined. Content validity and face validity were established by expert evaluation and focused group discussions.

Construct validity was established by exploratory factor analysis with varimax rotation to test the hypothesized domain structure and examine its substructure. Items with correlation coefficient > 0.7 were omitted. Internal consistency was examined, but test/retest reliability could not be performed because of paucity of time. The homogeneity of the question items in each domain was evaluated using Cronbach's α coefficient. A coefficient of 0.7 or higher is preferred for a questionnaire to be internally consistent.

Data analysis: data analysis done by the SPSS program version 23. Descriptive statistics in the form frequencies and percentages were used to describe the demographics; sleep characteristics and COVID-19 related knowledge among the study participants. Means and Standard deviations (mean ± SD) were used to represent continuous variable and Pearson’s Chi-Square test was used to test the association between categorical variables.

### Results

Of the participants, 340 (73.6%) had an age ranging from 23–30 years, 235 (50.9%) were females, 202 (43.7%) were from Taif city, 256 (55.4%) were residents, and 18.6% had an internal medicine specialty. The mean knowledge score in our study was found to be 4.23 ± 1.06 thus showing ‘good’ scores by 78.1% (n = 361), 12.1% showed fair knowledge and 9.7% gave poor scores. When the relationship of the knowledge scores and difference sociodemographic characteristics were assessed, there was no statistically significant difference observed with the gender, age, position, specialty and region of practice of doctors (Table 1, Additional file 1: Figure S1).

**Table 1:** Relationship of Knowledge about COVID-19 with gender, age and designation of participants

Variable		Knowledge			Total	X^2^	p value
		Good	Fair	Poor			
Gender							
Female	N	182	26	19	227	1.261	0.532
	%	80.2%	11.5%	8.4%	49.1%		
Male	N	179	30	26	235		
	%	76.2%	12.8%	11.1%	50.9%		
Age							
23–30 years		266	41	33	340	4.476	0.613
		78.2%	12.1%	9.7%	73.6%		
31–40 years		67	8	6	81		
		82.7%	9.9%	7.4%	17.5%		
41–50 years		12	4	2	18		
		66.7%	22.2%	11.1%	3.9%		
51–60 years		16	3	4	23		
		69.6%	13.0%	17.4%	5.0%		
Position							
Consultant	N	39	8	4	51	2.734	0.950
	%	76.5%	15.7%	7.8%	11.0%		
Associate consultant/Registrar	N	23	5	4	32		
	%	71.9%	15.6%	12.5%	6.9%		
Specialist	N	24	3	3	30		
	%	80.0%	10.0%	10.0%	6.5%		
Resident	N	205	28	23	256		
	%	80.1%	10.9%	9.0%	55.4%		
Intern	N	70	12	11	93		
	%	75.3%	12.9%	11.8%	20.1%		

The prevalence of sleep disorders in our study was found to be 43.9% during COVID-19 pandemic. When the relationship of sleep disorders with different sociodemographic and work-related characteristics was compared, there was significant association observed with age, position and specialty. Doctors who belonged to age group of 31–40 years showed more prevalence of sleep disorders (63%) compared to other age groups (p = 0.002). When the prevalence of sleep disorders with different position of doctors at hospitals was assessed, it was found that associate consultants and residents showed more sleep disorders than other groups with a highly statistically significant association. The prevalence of sleep disorders was found to be 53.1% and 49.6% in associate consultants and residents respectively (p < 0.001) (Table 2). When the prevalence of SD was examined based on different specialties, anesthetists, Laboratory/Pathology/Microbiology specialists and dentists had the highest prevalence (66.7%) compared to other specialties with a statistically significant association (p = 0.024) (Additional file 1: Figure S2).

**Table 2:** Prevalence of sleep disorders and its relationship with Age, gender and designation of participants

Variable	Sleep disorder		Total	*X*^*2*^	p value	
	Absent	Present				
Gender						
Female	N	123	104	227	0.637	0.425
	%	54.2%	45.8%	49.1%		
Male	N	136	99	235		
	%	57.9%	42.1%	50.9%		
Age						
23–30 years	N	204	136	340	14.445	0.002
	%	60.0%	40.0%	73.6%		
31–40 years	N	30	51	81		
	%	37.0%	63.0%	17.5%		
41–50 years	N	11	7	18		
	%	61.1%	38.9%	3.9%		
51-60 years	N	14	9	23		
	%	60.9%	39.1%	5.0%		
Position						
Consultant	N	26	25	51	22.200	<0.001
	%	51.0%	49.0%	11.0%		
Associate consultant/Registrar	N	15	17	32		
	%	46.9%	53.1%	6.9%		
Specialist	N	17	13	30		
	%	56.7%	43.3%	6.5%		
Resident	N	129	127	256		
	%	50.4%	49.6%	55.4%		
Intern	N	72	21	93		
	%	77.4%	22.6%	20.1%		

Assessment of different sleep characteristics during COVID-19 is given in Table 3. When the participants’ difficulty to fall asleep within 30 min was measured, it was found that 24.7% had the difficulty to fall asleep twice or more in a week during COVID-19, compared to 10.2% before the pandemic with a statistically significant difference (p < 0.001). Difficulty in fall asleep during COVID-19 showed a statistically significant association with specialty of the participants, where medical interns and doctors working in laboratory/pathology/microbiology department had significantly more difficulty (p = 0.002). The evaluation of usage of sleeping pills during COVID-19 showed that 3.9% and 10.2% used it three or more times a week and one or twice week respectively. This usage was more seen in internists (31.9%) and surgeons (19.1%) compared to other specialties with a statistically significant association (p = 0.028) (Table 3).

**Table 3:** Sleep characteristics and its relationship with Sociodemographic characters

Variable	N	%	Gender	Age	Position	Specialty
Difficulty to fall asleep within 30 minutes during the COVID-19						
Less than once a week	108	23.4	0.066	0.221	0.471	0.002
Not during the past two months	130	28.1				
Once or twice a week	110	23.8				
Three or More times a week	114	24.7				
Difficulty to fall asleep within 30 minutes before the COVID-19						
Less than once a week	114	24.7	0.588	0.518	0.022	0.115
Not at all	189	40.9				
Once or twice a week	112	24.2				
Three or More times a week	47	10.2				
Taking pills to get sleep						
Less than once a week	79	17.1	0.569	0.045	0.075	0.028
Not during the past two months	318	68.8				
Once or twice a week	47	10.2				
Three or More times a week	18	3.9				
Trouble staying awake while driving, eating meals or engaging in social activity						
Less than once a week	96	20.8	0.069	0.195	0.002	0.087
Not during the past two months	274	59.3				
Once or twice a week	64	13.9				
Three or More times a week	28	6.1				
Sleep duration during COVID-19						
< 6 h	176	38.1	0.211	0.097	0.000	0.009
6–8 h	232	50.2				
> 8 h	54	11.7				
Sleep duration before COVID-19						
< 6 h	149	32.3	0.083	0.185	0.320	0.021
6–8 h	266	57.6				
> 8 h	47	10.2				
Sleep quality during the pandemic						
Very good	108	23.4	0.027	0.113	0.455	0.056
Fairly good	277	60.0				
Fairly bad	61	13.2				
Very bad	16	3.5				

The prevalence of trouble in staying awake while driving, eating meals or engaging in social activity was 6.1% and 13.9% in ≥ 3 times a week and once or twice week respectively. This was significantly more reported in resident doctors (≥ 3 times/week = 71.4% and once/twice = 68.8%) compared to other groups (p = 0.002). When sleep duration among doctors was measured, 38.1% reported sleeping for < 6 h/day during COVID-19 compared to 32.3% before COVID-19 with a statistically significant difference (p < 0.001). Residents (45.7%) and consultants (43.1%) showed a significant higher percent of those who suffered decreased sleep duration (< 6 h) during COVID-19 (p < 0.001).

The overall sleep quality during COVID-19 was observed to be very good and fair good in 23.4% and 60% of doctors respectively. Only 3.5% of the doctors were found to have ‘very bad’ sleep during this pandemic, and this was significantly more prevalent in female doctors (5.3%) compared to males (1.7%) (p = 0.027).

### Discussion

COVID-19 pandemic is a severe challenge for healthcare physicians in the Kingdom of Saudi Arabia (KSA) [15]. Doctors are at risk of getting infected and are often under great stress thinking of the possible transmission of the infection to their family members, colleagues, and other patients [15]. Epidemiological studies done in the kingdom have reported a very high prevalence of poor sleep quality among the Saudi adult population [16, 17]. In addition to a poor sleep quality among physicians with a prevalence of 50% or more [18, 19]. COVID-19 has affected the mental wellbeing of health care workers that might have an impact on sleep quality [20].

The present study demonstrated that 78.1% of the doctors had good knowledge regarding COVID-19 irrespective of age, gender, position, and specialty. Another study demonstrated insufficient knowledge among doctors about this pandemic [21]. An excellent knowledge about COVID-19 is very essential among physicians when managing or treating patients [22].

The prevalence of sleep disorders among participants of this study was 43.9%. A study conducted in Iraq reported a prevalence of sleepless of 68.3% [23]. Good quality sleep is very much essential to boost the immunity that would help to fight against the viruses and disease [24], and to improve doctor-patient relationship [25].

In the present study physicians had more difficulty falling asleep during COVID-19. Impaired sleep due to high work demands, irregular break-times, and stress may cause clinical burnout in healthcare workers [26]. Sleep disorders were significantly more in physicians who had an age ranging from 31–40 years. This finding is consistent with another study [27]. Also, it was found that residents and the associate consultants had significantly more sleep disorders than others. Young physicians especially residents or associate consultants may be experiencing such a pandemic for the first time, this may force them to sleep in the hospital and lead to sleep deprivation.

The study findings also showed that the trouble staying awake while driving, eating meals, or engaging in social activity was found to be more in resident doctors. Previous studies reported that that physicians who had sleep deprivation are at increased risk of facing motor accidents while driving [28, 29].

The prevalence of sleep disorder during COVID-19 was high among physicians belong to emergency medicine, anesthesia, and Laboratory/Pathology/Microbiology. It is somehow clear that doctors working in laboratories have been working diligently during the last few months giving more priorities for the COVID-19 tests than other routine tests [30]. Sleep quality assessment showed that female physicians had poor sleep quality compared to males. A result revealed from a previous study [30]. Thus, risks of committing medical errors in physicians who have sleep deprivation are more due to cognitive and motor impairment [31, 32]. All these problems arise from a shortage of staff in the respective departments that force the doctors to work for prolonged hours than their actual work hours [33]. During a pandemic, the shortage of medical staff may become worse as some doctors may abstain from work due to fear of getting infected [33].

The hospital administrations should provide optimal work hours with enough break or leisure time, employing more doctors during this pandemic, monitor the doctor-patient ratio, educational and training programs to reduce stress among doctors.

## Limitations

The study didn't check the influence of many factors as total working hours/week and substances abuse. The self-reporting questionnaire may have a recall bias.

## Supplementary information

**Additional file 1: Figure S1.** Knowledge about COVID-19 among different specialities.** Figure S2.** Prevalence of sleep disorders according to specialties.

## Data Availability

The datasets used and/or analysed during the current study are available from the corresponding author on reasonable request.

## Abbreviations

SARS: Severe Acute Respiratory Syndrome
MERS-CoV: Middle East Respiratory Syndrome Coronavirus
PSQI: Pittsburgh Sleep Quality Index scale
SD: Standard deviation
KSA: Kingdom of Saudi Arabia
