# Supplementary material for: Sleep disturbances among physicians during COVID-19 pandemic
Source: BMC Res Notes. 2020 Oct 21;13:493. doi: 10.1186/s13104-020-05341-6 (PMC7576978; doi:10.1186/s13104-020-05341-6)

**Figure S1. Knowledge about COVID-19 among different specialities**

**
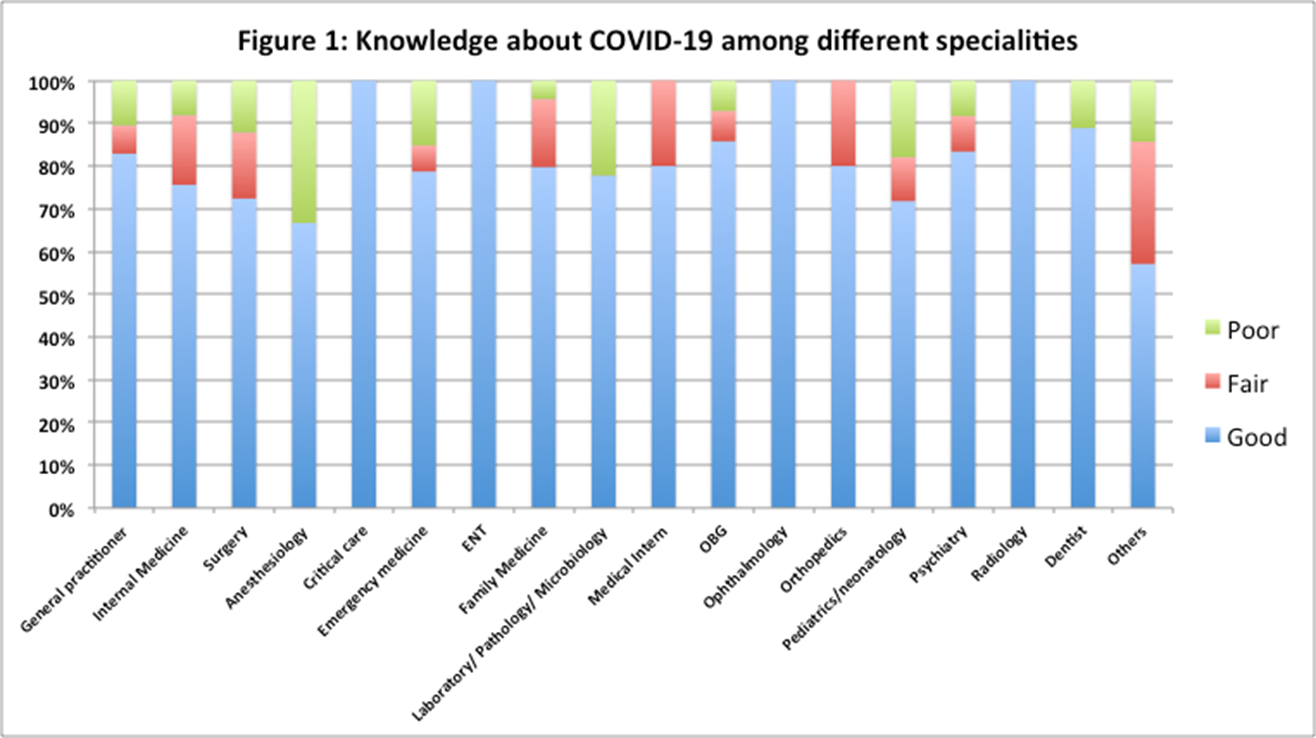
**

**Figure S2. Prevalence of sleep disorders according to specialties**


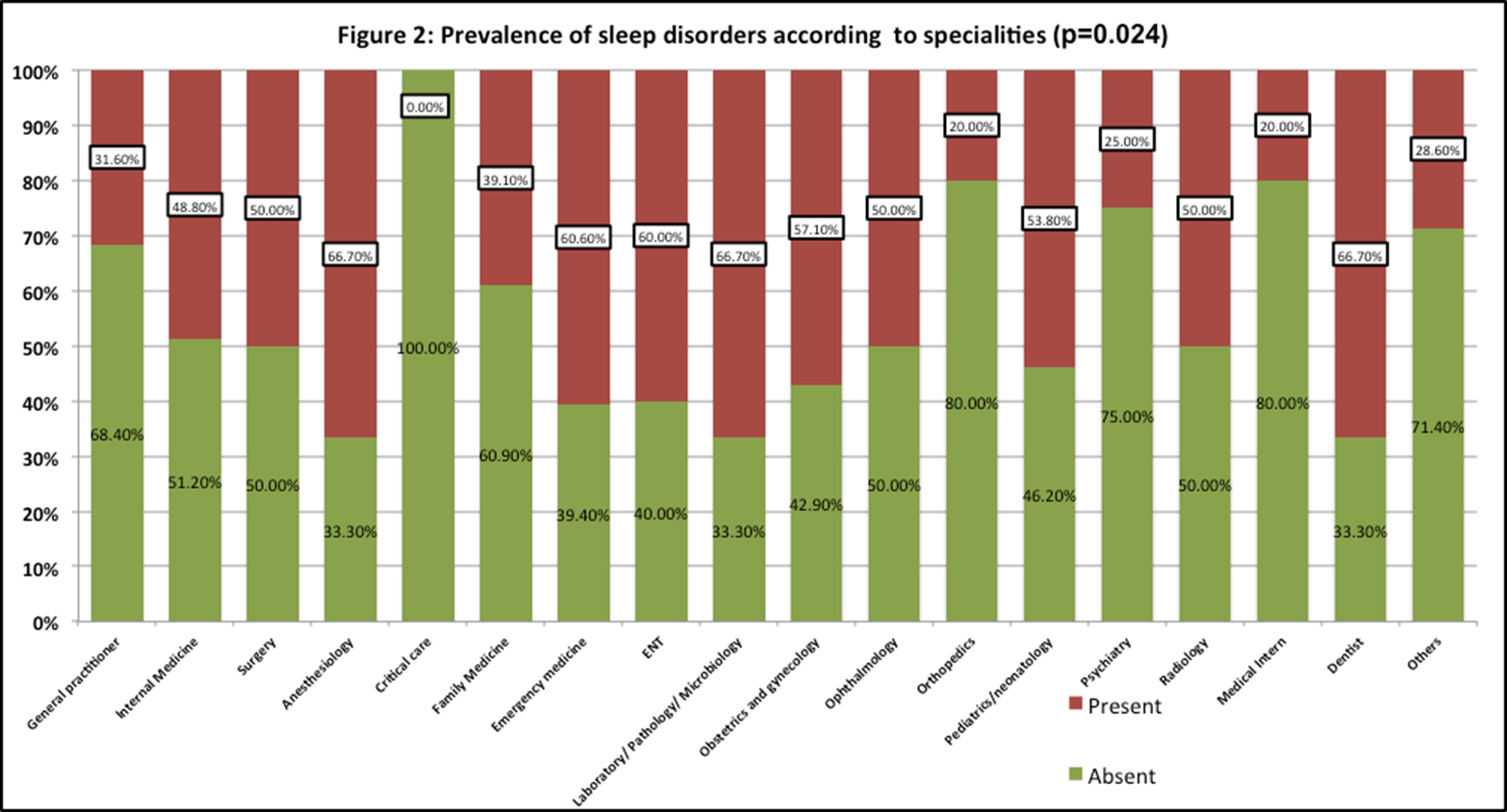

Supplement: Supplementary file 1 — Additional file 1: Figure S1. Knowledge about COVID-19 among different specialities. Figure S2. Prevalence of sleep disorders according to specialties. [file 13104_2020_5341_MOESM1_ESM.docx]
